# Association of Affordable Care Act Implementation With Ambulance Utilization for Asthma Emergencies in New York City, 2008-2018

**DOI:** 10.1001/jamanetworkopen.2020.25586

**Published:** 2020-11-11

**Authors:** Gregory A. Peters, Alexander J. Ordoobadi, Rebecca E. Cash, Matthew L. Wong, Paul Avillach, Carlos A. Camargo

**Affiliations:** 1 Department of Emergency Medicine, Massachusetts General Hospital, Harvard Medical School, Boston, Massachusetts; 2 Department of Emergency Medicine, Brigham and Women’s Hospital, Harvard Medical School, Boston, Massachusetts; 3 Department of Surgery, Brigham and Women’s Hospital, Boston, Massachusetts; 4 Department of Emergency Medicine, Beth Israel Deaconess Medical Center, Harvard Medical School, Boston, Massachusetts; 5 Department of Biomedical Informatics, Harvard Medical School, Boston, Massachusetts; 6 Department of Epidemiology, Harvard T.H. Chan School of Public Health, Boston, Massachusetts

## Abstract

**Importance:**

Emergency medical services (EMS) are an essential component of the health care system, but the effect of insurance expansion on EMS call volume remains unclear.

**Objective:**

This study investigated the association between health insurance expansion and EMS dispatches for asthma, an ambulatory care–sensitive condition. We hypothesized that insurance expansion under the Patient Protection and Affordable Care Act (ACA) would be associated with decreased EMS dispatches for asthma emergencies.

**Design, Setting, and Participants:**

This cohort study examined 14 865 267 ambulance calls dispatched within New York City from 2008 to 2018, including 217 303 calls for asthma-related emergencies, and used interrupted time series analysis to study the change in the annual incidence of EMS dispatches for asthma emergencies after implementation of the ACA. Multivariable linear regression examined the association between the uninsured rate and the incidence of asthma-related dispatches, controlling for population demographic characteristics and air quality index.

**Exposures:**

Implementation of ACA on January 1, 2014.

**Main Outcomes and Measures:**

Incidence of EMS dispatches for asthma emergencies per 100 000 population per year (ie, asthma EMS dispatch rate) as classified by the 911 call–taker.

**Results:**

In this study of 217 303 EMS dispatches for asthma-related emergencies, there was a decrease in the asthma EMS dispatch rate after implementation of the ACA, from a mean (SD) of 261 (24) dispatches per 100 000 population per year preintervention to 211 (47) postintervention (*P* = .047). This decrease in asthma EMS dispatch rate after ACA implementation was significant on interrupted time series analysis. Prior to 2014, the annual asthma EMS dispatch rate was increasing by 11.8 calls per 100 000 population per year (95% CI, 6.1 to 17.4). After ACA implementation, the asthma EMS dispatch rate decreased annually by 28.5 calls per 100 000 population per year (95% CI, −37.6 to −19.3), a significant change in slope from the preintervention period (*P* < .001). Multivariable linear regression, controlling for percentage of individuals younger than age 18 years, degree of racial/ethnic diversity, median household income, and air quality index, found that a 1% decrease in the citywide uninsured rate was associated with a decrease of 98.9 asthma dispatches per 100 000 population per year (95% CI, 5.72-192.10; *P* = .04).

**Conclusions and Relevance:**

Insurance expansion within New York City under the ACA was associated with a significant reduction in the asthma EMS dispatch rate. Insurance expansion may be a viable method to reduce EMS utilization for ambulatory care–sensitive conditions such as asthma.

## Key Points

**Question:**

What is the association between insurance expansion and emergency medical services (EMS) dispatches for an ambulatory care–sensitive condition like asthma?

**Findings:**

In this cohort study including 217 303 EMS dispatches for asthma emergencies in New York City, implementation of the Patient Protection and Affordable Care Act was associated with a decrease in calls for asthma emergencies. In adjusted models, larger decreases in the uninsured rate were associated with larger decreases in the asthma EMS dispatch rate.

**Meaning:**

The findings of this study suggest that insurance expansion may lead to improved outpatient management of ambulatory care–sensitive conditions like asthma, resulting in decreased utilization of EMS.

## Teaser

This quasi-experimental cohort study assesses the association between Affordable Care Act implementation and asthma treatment by examining changes in ambulance dispatch rates for asthma emergencies in New York City between 2008 and 2018.

## Introduction

Emergency medical services (EMS) in the US respond to millions of calls each year,^1^ delivering prehospital care to patients with conditions ranging from life-threatening emergencies to minor injuries. Most EMS systems have fixed resources, including a limited number of personnel and ambulances. A strain on these systems because of increased utilization or other factors can result in delayed care and even increased mortality for patients with serious emergencies.^2,3,4,5^ For this reason, changes in EMS utilization, such as those triggered by health insurance changes, could have important implications for EMS systems.

Prior studies of the effects of health insurance expansion on emergency services utilization have yielded conflicting results.^6^ Insurance expansion, including through the 2014 implementation of the Patient Protection and Affordable Care Act (ACA), was shown in some studies to increase the number of visits to emergency departments.^7,8,9^ Similarly, ACA implementation was associated with an increase in EMS utilization for minor injuries in New York City (NYC).^10^ However, other studies^11,12,13,14^ have demonstrated no change after insurance expansion or even a decrease in utilization of emergency services. This reduction in utilization could be driven by improved management of ambulatory care–sensitive conditions, a category of chronic diseases that can result in emergency department visits if not properly controlled through primary care.^15^ Asthma has been extensively studied as an example of an ambulatory care–sensitive condition, and lack of insurance is a risk factor for developing asthma exacerbations that require emergency care.^16,17,18,19^ However, it is unknown whether EMS utilization for ambulatory care–sensitive conditions would decrease due to the assumed improvement in primary care management related to insurance expansion.

To investigate the association of insurance expansion with EMS utilization for ambulatory care–sensitive conditions, we studied ambulance dispatches for asthma emergencies within the NYC EMS system from 2008 through 2018 (ie, before and after implementation of the ACA in 2014). Because of its high prevalence, asthma is a particularly important disease to study in NYC.^20^ Furthermore, asthma can be rapidly controlled through primary care interventions, including improved maintenance and rescue therapy, meaning that expanded access to primary care could in theory reduce asthma exacerbations within the time scale of this study. An assessment of the association of health policy changes with ambulance utilization for a highly prevalent disease in a high-volume EMS system could provide valuable insights for public health. We hypothesized that insurance expansion under the ACA would be associated with decreased EMS dispatches for asthma emergencies.

## Methods

### Study Design, Data Sources, and Setting

We performed a cohort study using interrupted time series analysis in addition to linear modeling to study the association between insurance expansion under the ACA and EMS dispatches for asthma emergencies. The primary data source was a publicly available database of EMS incident dispatch data encompassing every 911 call resulting in an ambulance dispatch within NYC from January 1, 2008, to December 31, 2018.^21^ NYC has a centralized 911 dispatch system that captures every 911 call within city limits regardless of the responding agency.^22^ This system ensures that all EMS calls are captured within the data set. The data sets and analytic plan were reviewed by the Harvard Longwood Campus institutional review board and deemed exempt from informed consent requirements because all data included in this analysis are freely available to the public. This study followed the Strengthening the Reporting of Observational Studies in Epidemiology (STROBE) reporting guideline.^23^

The computer-aided dispatch system used in NYC includes asthma as a distinct call type category, as opposed to other systems that may not differentiate between respiratory concerns.^24^ The specific dispatch codes used by the Fire Department of New York that we classified as asthma included ASTHFC (asthma attack associated with fever and cough), ASTHFT (asthma attack associated with fever and positive travel history), ASTHMA (asthma attack), ASTHMB (asthma attack), and ASTHMC (asthma attack associated with critical condition). For comparison with a nonambulatory care–sensitive condition, we repeated this procedure on all EMS dispatches classified as STAB to indicate stabbings throughout the study period. Because this data set only contains deidentified EMS incident dispatch data, individual patient demographic information including age, sex, race, and insurance status are not available.

The EMS dispatch data set was enriched with several other population-level data sets. The zip code of each incident location was used to link each dispatch with local estimates of population size, percentage of individuals without health insurance coverage, percentage of individuals under age 18 years, degree of racial/ethnic diversity (defined as the percentage of zip code residents who identify as non-Hispanic White only), median household income, and air quality index (AQI). These variables were compiled from 5-year estimates generated by annual American Community Survey (ACS) data from 2012 to 2018 (where the 5-year estimates from 2012 were applied to 2008-2012),^25^ with the exception of daily AQI estimates, which were provided by the US Environmental Protection Agency (EPA).^26^ ACS data were organized both at the county (ie, borough) level to compute citywide estimates weighted by county population, and at the zip code level for greater geographical resolution. Although ACS data would accommodate even greater geographical resolution, such as at the census tract level of analysis, the publicly available EMS dispatch data used for this study does not specify location beyond zip code in order to maintain the privacy of deidentified patients. Daily AQI measurements within each borough were averaged together and assigned to each corresponding zip code. In the absence of EPA measurements of AQI in Brooklyn, we used measurements from the nearest site in Queens.

Once compiled, this composite data set was used to compute estimates of asthma-related emergency incidence at the citywide and zip code levels. At the zip code level, the analysis was restricted to the 168 zip codes in NYC with a population greater than 10 000 as of 2018. Given that the ACA was implemented on January 1, 2014, we designated 2008 to 2013 as the preintervention period and 2014 to 2018 as the postintervention period.

### Statistical Analysis

Descriptive statistics were calculated for NYC, and comparisons of measures pre-ACA and post-ACA implementation were made using 2-tailed *t* tests. The primary outcome measure was the asthma EMS dispatch rate, which is the rate of ambulance dispatches for asthma emergencies per 100 000 population per year. We conducted an interrupted time series analysis of the annual citywide asthma EMS dispatch rate. As a sensitivity analysis, we conducted an interrupted time series analysis using all EMS dispatches over the same time period to determine if the changes observed were associated with underlying trends. We also repeated the interrupted time series analysis for stabbings as an example of a nonambulatory care sensitive condition. Stabbings were chosen because such incidents are easily identifiable, have been recorded as a discrete call type throughout the full study period, and are less likely to be confounded by other factors (eg, gunshot wound incidence can be affected by changes in firearm control legislation).

To further evaluate patterns in asthma EMS dispatch rate, we examined the association between uninsured rate and citywide asthma EMS dispatch rate using linear regression models, controlling for the population-level factors of percentage of individuals under age 18 years, degree of ethnic and racial diversity, median household income, and AQI. These variables were chosen from prior literature as potential confounders.^27,28,29,30,31^ Ordinary least-squares regression models were fit with Newey-West standard errors to adjust for autocorrelation and heteroskedasticity.^32,33^ After fitting ordinary least-squares models, we used the Cumby-Huizinga test^34^ for autocorrelation and included the appropriate lag term as indicated. Models allowed for both a trend (ie, slope) and level (ie, intercept) change post-ACA implementation. We repeated the linear regression models at the zip code level to test whether this association would hold across the 168 zip codes that compose NYC while controlling for other key factors. We fit generalized estimating equations to handle clustering by zip code, assuming working exchangeable correlation structure and robust standard errors. This analysis included a greater number of data points given its inclusion of space in addition to time in order to accommodate the inclusion of our 5 covariates; the models controlled for the same factors, which were also included at the zip code, rather than citywide, level. Changes in uninsured rate by zip code and asthma EMS dispatch rate were mapped to visually compare the relationship between these 2 variables. For all analyses, *P* < .05 was considered statistically significant. All analyses were performed in Stata version 15.0 (StataCorp) and R version 3.6.2 (R Foundation for Statistical Computing). Tableau Desktop version 2019.3 (Tableau Software) was used to graph zip code–level data on a crowdsourced web map produced by OpenStreetMap.^35^

## Results

A total of 14 865 267 EMS calls were dispatched during the study period, including 217 303 (1.5%) calls for asthma-related emergencies. The overall incidence of total EMS dispatches per 100 000 population per year at the citywide level increased during the study period from a mean (SD) of 15 471 (413) prior to ACA implementation to 17 143 (685) postimplementation (*P* < .001) (Table 1). After ACA implementation, there was a significant decrease in the asthma EMS dispatch rate (mean [SD], 261 [24] vs 211 [47] per 100 000 population per year; *P* = .047).

**Table 1.** EMS Dispatch Data and Population Demographic Characteristics Before and After Implementation of the ACA at the Citywide Level

Characteristics	Mean (SD)		*P* value^a^
	Pre-ACA	Post-ACA	
Years, No.	6	5	
EMS dispatches, per 100 000 population per y			
Total calls	15 471 (413)	17 143 (685)	<.001
Asthma calls	261 (24)	211 (47)	.047
Population demographic information			
Total population	8 210 851 (28 487)	8 449 476 (73 966)	<.001
Uninsured, %^b^	14.2 (0.07)	11.0 (2.03)	.003
Age <18 y, %^b^	21.6 (0.06)	21.1 (0.19)	<.001
Non-Hispanic White, %^b^	44.4 (0.09)	43.1 (0.40)	<.001
Median household income, $	52 733.85 (213.47)	57 214.49 (3262.76)	.008
Air Quality Index (unitless)	32.9 (2.55)	33.9 (1.19)	.44

Abbreviations: ACA, Affordable Care Act; EMS, emergency medical services.

^a^ *P* value calculated from *t* tests.

^b^ As percentage of total population.

There were several changes in population demographics within NYC during the study period at the citywide level (Table 1). The uninsured rate decreased after ACA implementation from 14.2% to 11.0% (−3.2%; 95% CI, −5.1% to −1.4%; *P* = .003). Median income increased from $52 734 to $57 214 ($4480; 95% CI, $1493-$7468.20; *P* = .008), while there were slight decreases in the percentage of the population under age 18 years (−0.48%; 95% CI, −0.67% to −0.29%; *P* < .001) and the percentage of the population who were non-Hispanic White individuals (−1.3%; 95% CI, −1.7% to −0.97%; *P* < .001). There was no significant change in the level of air pollution in the city, as represented by the AQI (1.0%; 95% CI, −3.8 to 1.8; *P* = .44).

**Figure 1.**
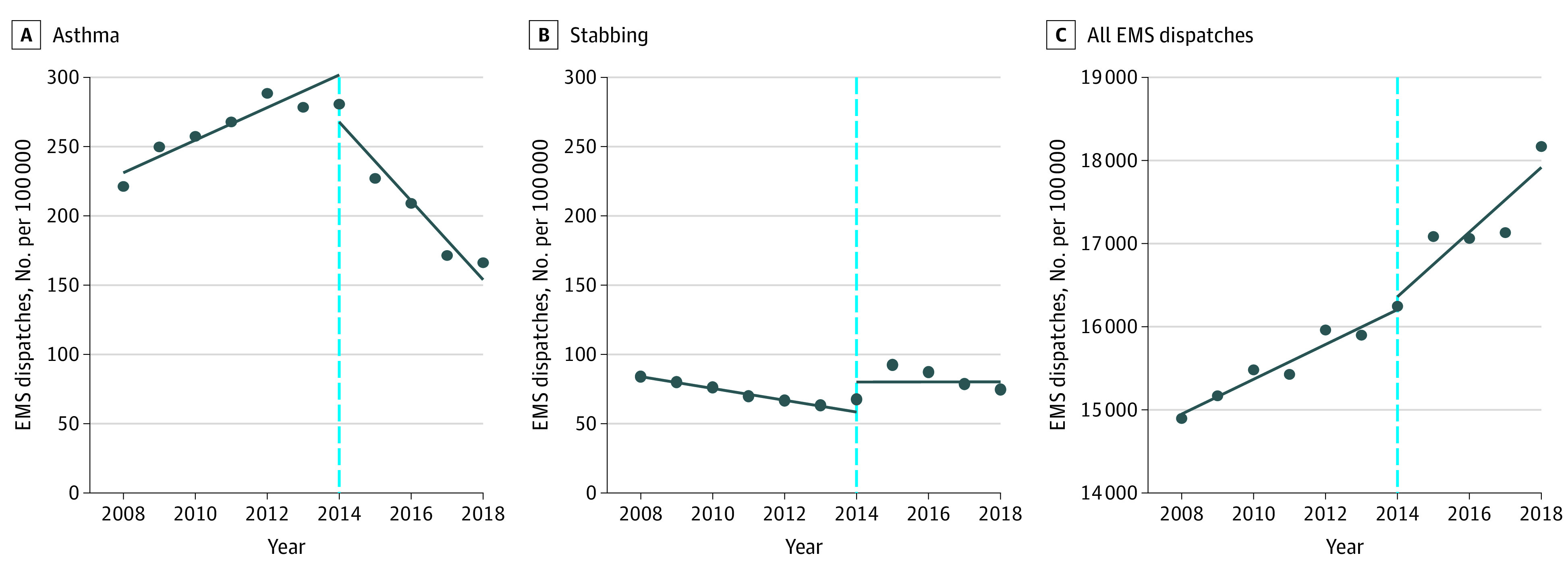
Interrupted Time Series Analysis of EMS Dispatches in New York City at the Citywide Level Before and After Implementation of Insurance Expansion on January 1, 2014 Abbreviations: EMS, emergency medical services.

Interrupted time series analysis demonstrated a significant decrease in the incidence of asthma dispatches after ACA implementation at the citywide level (Figure 1). Prior to 2014, the annual asthma EMS dispatch rate was increasing by 11.8 calls per 100 000 population per year (95% CI, 6.1 to 17.4). After ACA implementation, the asthma EMS dispatch rate decreased annually by 28.5 calls per 100 000 population per year (95% CI, −37.6 to −19.3), a significant change in slope from the preintervention period (*P* < .001). By contrast, there was a significant increase of all EMS dispatches following ACA implementation (change in slope, 180; 95% CI, 57 to 302; *P* = .01). The EMS dispatch rate for stabbings, a nonambulatory care–sensitive condition, did not change significantly after implementation of the ACA (change in slope, 4.3; 95% CI, −4.6 to 13.2; *P* = .29).

Unadjusted linear regression analysis revealed a significant positive association between the citywide annual uninsured rate and asthma EMS dispatch rate (coefficient, 17.8; 95% CI, 10.6 to 25.0; *P* < .001), with an adjusted *r^2^* = 0.75. This association persisted on multivariable linear regression analysis, where a 1% decrease in the citywide uninsured rate was associated with a mean decrease of approximately 99 asthma EMS dispatches per 100 000 population per year (98.91; 95% CI, 5.72-192.10; *P* = .04) (Table 2).

**Table 2.** Multivariable Linear Regression Model for Asthma Emergency Medical Services Dispatch Rate at the Citywide Level

Covariate	Coefficient (95% CI)^a^	*P* value
Uninsured rate, %	98.91 (5.72 to 192.10)	.04
Age <18 y, %	41.23 (−477.63 to 560.09)	.85
Non-Hispanic White, %	−75.46 (−265.36 to 114.44)	.35
Median household income, $1000	40.48 (−16.22 to 97.30)	.13
Air quality index	6.27 (−1.57 to 14.11)	.10

^a^ Coefficients describe the rise in asthma-related EMS dispatches per 100 000 population per year, per the rise of a single unit in each covariate.

**Figure 2.**
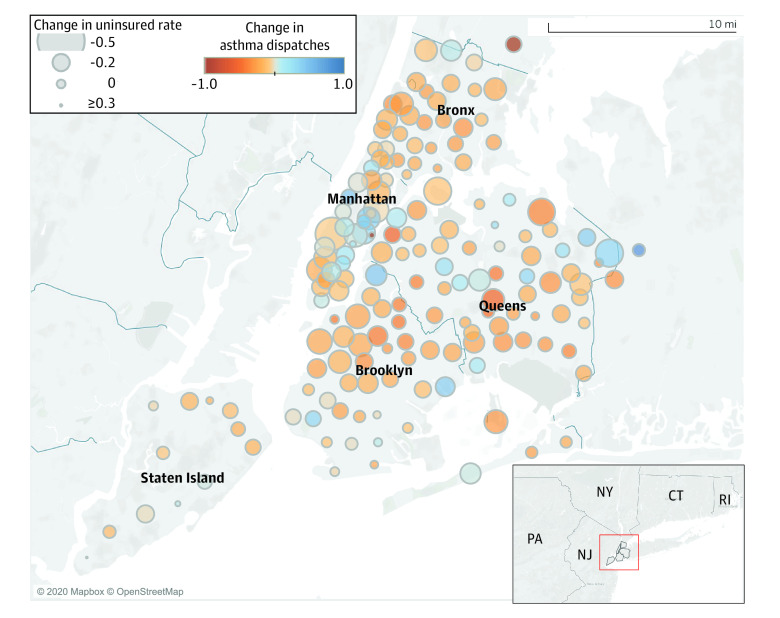
Geographical Analysis Comparing Change in Uninsured Rate and Change in Rate of Asthma Dispatches Within Zip Codes in New York City Larger circles indicate greater reduction in uninsured rate, while orange color indicates greater reduction in rate of asthma dispatches.

We repeated the multivariable linear regression model at the zip code-level. This model showed a similar significant and positive association between uninsured rate and asthma EMS dispatch rate, where a 1% decrease in the uninsured rate was associated with a mean decrease of 10.25 asthma dispatches per 100 000 population per year (95% CI, 7.26-13.23; *P* < .001) (Table 3). Finally, in the geographic analysis based on zip codes within NYC, geographical overlap was noted between regions of the city with greater reductions in uninsured rate and regions with reductions in the asthma EMS dispatch rate (Figure 2). Zip codes within the Bronx and most of Brooklyn had the most consistent decreases in both the uninsured rate and the asthma EMS dispatch rate.

**Table 3.** Zip Code–level Sensitivity Analysis From a Generalized Estimating Equation Model for Rate of Emergency Medical Services Dispatches for Asthma Emergencies

Covariate	Coefficient (95% CI)^a^	*P* value
Uninsured rate	10.25 (7.26 to 13.23)	<.001
Age <18 y	9.41 (3.62 to 15.20)	.001
Non-Hispanic White	−0.58 (−2.44 to 1.27)	.54
Median household income, $1000	−1.11 (−2.30 to 0.08)	.07
Air quality index	1.05 (0.27 to 1.83)	.008

^a^ Coefficients describe the rise in asthma-related EMS dispatches per 100 000 population per year, per the rise of a single unit in each covariate.

## Discussion

In this cohort study of EMS dispatches for asthma emergencies in NYC, the asthma EMS dispatch rate significantly decreased in the years after implementation of the ACA. By comparison, the EMS dispatch rate for stabbings, a nonambulatory care–sensitive condition, remained unchanged. There was also an increase in EMS utilization overall during the study period, which mirrors an ongoing national trend of increasing EMS transports.^36,37^ The decrease in the asthma EMS dispatch rate was significantly associated with annual citywide decreases in the uninsured rate, which were most pronounced in the years following implementation of the ACA. Furthermore, this association persisted after controlling for age, race/ethnicity, median household income, and AQI. The citywide association between the decreased uninsured rate and decreased asthma EMS dispatch rate also held at the zip code level after controlling for local estimates of the same set of key factors. Therefore, our findings consistently demonstrate an association between insurance expansion delivered by the ACA and decreasing asthma EMS dispatch rate in NYC.

The mechanism by which health insurance expansion is associated with fewer asthma-related emergencies severe enough to require EMS response is most likely related to improved outpatient management of the disease. Asthma is a chronic medical condition that is associated with acute exacerbations if not properly controlled.^38^ Because effective chronic care for patients with asthma can decrease exacerbations,^39,40^ increased access to primary care through insurance expansion is likely to improve the control of asthma and reduce exacerbations. The ways in which insurance expansion for asthma patients can reduce emergency services utilization include improved control therapy (such as inhaled corticosteroids) to reduce exacerbations; improved rescue therapy (such as inhaled beta-agonists) to de-escalate exacerbations; reduced cost of these medications; improved follow-up after an exacerbation^41,42,43^; and increased access to counseling regarding the avoidance of triggers, proper medication administration, and the formulation of an asthma action plan.^44,45,46,47^ Both missed primary care appointments and gaps in insurance are associated with increased emergency department utilization among children with asthma.^17,48^ Together, these prior findings provide possible mechanisms for the reduction in EMS activations for asthma within NYC in the years after implementation of the ACA.

In contrast to our results, 2 previous studies by Courtemanche et al^2,10^ reported that ACA implementation was associated with increased EMS dispatches within the NYC EMS system. The first showed that ambulance dispatches for minor injuries increased following implementation of the ACA,^10^ and the second described an overall increase in all calls of relatively lower severity over the same time period.^2^ The authors attributed these trends to insurance expansion on the grounds that health insurance insulated patients from the costs of utilizing EMS.^10^ Unlike asthma, minor injuries are not sensitive to ambulatory care, so increased access to primary care services would not have had an effect on the incidence of minor emergencies. We therefore believe that increased ambulance utilization for minor injuries does not have any bearing on our explanations for the asthma findings. Moreover, our finding that the decreased asthma EMS dispatch rate was associated not just with the implementation of ACA in 2014 but also with insurance expansion itself on an annual basis (as opposed to other changes that might have coincided with 2014) further supports our conclusion that improved access to care may have contributed to lower rates of asthma emergencies in NYC.

### Limitations

This study has several potential limitations. Asthma emergencies were identified based on dispatch information rather than on a clinician’s assessment of the patient. However, inaccuracies in assessing the patient’s chief concern over the telephone are unlikely to have changed from year to year or based on insurance status. In addition, there have been changes in EMS dispatch call classification algorithms used by the Fire Department of New York during the study period, such as during the implementation of a new computerized triage system in 2017.^49^ However, we are not aware of relevant changes in classification procedures that coincided with 2014. Even if such changes did occur, they would not directly affect our analyses that examine uninsured rates rather than year as the primary independent variable, and which revealed the same finding. Moreover, such policy changes would be implemented citywide and would therefore not affect our analyses at the zip code level. Another limitation is that individual patients may be captured multiple times within the EMS dispatch data set, resulting in nonindependence. In addition, the zip code of each EMS incident is not necessarily the zip code in which the patient resides, which may result in misclassification of covariates. It is also possible that other interventions took place in 2014 besides the implementation of ACA that could have contributed to the change in ambulance dispatches observed on interrupted time series analysis. However, we have not identified any public health or policy measures dedicated to controlling asthma that were implemented within NYC around 2014. Moreover, it is possible that changes in EMS utilization might not necessarily reflect changes in emergency department utilization. Future studies should be completed to evaluate whether these trends can be observed in emergency department utilization for asthma in NYC, as well as whether similar trends can be observed for the broader category of all respiratory complaints. Finally, it is important to note that there are inherent limitations in using interrupted time series analysis to make causal claims, so we can only demonstrate an association between insurance expansion under the ACA and a decrease in the asthma EMS dispatch rate.

## Conclusions

In summary, EMS is an important component of the health care system, but there is a paucity of studies on the effects of health policy decisions on EMS systems. This study demonstrates the ability to use EMS administrative data to study the implications of policy decisions on EMS utilization. Whereas single hospital site or system data sets do not capture incidents presented to external institutions, and whereas insurance claims data sets only capture incidents among their particular patient cohort (which can skew the sample and limit generalizability), EMS administrative data sets can provide a full assessment of acute care utilization patterns at a population level within a particular geographic area. For policymakers, the study supports the growing body of evidence that insurance expansion improves the control of ambulatory care–sensitive conditions such as asthma, as shown by significant decreases in the utilization of emergency services for these conditions. It also illustrates that the association of insurance expansion with EMS call volume may vary depending on the type of emergency. Ambulatory care–sensitive conditions should be targeted by policy makers hoping to reduce EMS utilization through insurance expansion.
